# Unveiling Novel Molecular Candidate Targets of Loratadine, Raloxifene, and Sorafenib in Hepatocellular Carcinoma

**DOI:** 10.3390/ijms27198648

**Published:** 2026-09-27

**Authors:** Fernanda Villarruel-Melquiades, Martha Noyola-Díaz, Gelacio Martínez-Gudiño, María Eugenia Mendoza-Garrido, José Rubén García-Sánchez, Cecilia Bañuelos, Julio Isael Pérez-Carreón, Javier Camacho

**Affiliations:** 1 Departamento de Farmacología, Centro de Investigación y de Estudios Avanzados del Instituto Politécnico Nacional, Av. Instituto Politécnico Nacional 2508, Ciudad de México 07360, Mexico; junoyola@cinvestav.mx (M.N.-D.); gmartinezg@cinvestav.mx (G.M.-G.); 2 Departamento de Fisiología, Biofísica y Neurociencias, Centro de Investigación y de Estudios Avanzados del Instituto Politécnico Nacional, Av. Instituto Politécnico Nacional 2508, Ciudad de México 07360, Mexico; mmendoza@fisio.cinvestav.mx; 3 Sección de Estudios de Posgrado e Investigación, Escuela Superior de Medicina del Instituto Politécnico Nacional, Plan de San Luis y Díaz Mirón, Ciudad de México 11340, Mexico; jrgarcias@ipn.mx; 4 Departamento de Investigación y Estudios Multidisciplinarios, Programa Transdisciplinario en Desarrollo Científico y Tecnológico para la Sociedad, Centro de Investigación y de Estudios Avanzados del Instituto Politécnico Nacional, Av. Instituto Politécnico Nacional 2508, Ciudad de México 07360, Mexico; cebanuelos@cinvestav.mx; 5 Instituto Nacional de Medicina Genómica (INMEGEN), Periférico Sur No. 4809, Col. Arenal Tepepan, Ciudad de México 14610, Mexico; jiperez@inmegen.gob.mx

**Keywords:** hepatocellular carcinoma, drug repurposing, DARTS assay, loratadine, raloxifene, sorafenib, drug targets

## Abstract

Hepatocellular carcinoma (HCC) remains a major global health burden. We have recently demonstrated the synergistic cytotoxicity elicited by the combination of loratadine, raloxifene, and sorafenib in liver cancer cells. In this study, we investigated the potential mechanistic basis of this novel multi-drug regimen by identifying candidate protein targets of the three drugs. Potential protein targets of each drug were identified using the Drug Affinity Responsive Target Stability (DARTS) assay in protein extracts from the HCC cell line HuH7, and molecular docking was performed to predict binding modes and explore the structural feasibility of these drug–target interactions. Bioinformatics analyses were conducted to predict biological functions, molecular interactions, and clinical relevance. We identified 147 potential candidate DARTS-stabilized proteins for loratadine, 191 for sorafenib, and 157 for raloxifene; a proportion of these candidate proteins maintained a statistically significant association with overall survival in HCC patients: 20.14% for loratadine, 22.93% for raloxifene, and 24.47% for sorafenib. Cross-referencing with public databases confirmed several previously documented targets—complementing our experimental approach—while revealing numerous previously unreported target candidates. Docking analyses predicted favorable binding interactions between the drugs and several proteins, such as the putative complex between loratadine and N-glycanase 1 (NGLY1), whose expression correlates with reduced overall survival in HCC patients. Functional enrichment analysis showed that several targets are involved in hallmarks of cancer. Clinically relevant candidate targets of loratadine, raloxifene, and sorafenib were identified. However, as these candidate protein targets were identified exclusively in HuH7 cells, they may not fully generalize across all HCC models without further validation. Nevertheless, our results suggest that loratadine, raloxifene and sorafenib modulate key pathways involved in tumorigenesis. These findings strengthen the use of this combination as a promising therapeutic strategy for HCC and provide new opportunities for the identification of novel targets, prognostic markers, and molecular vulnerabilities suitable for pharmacological intervention in HCC and other malignancies and diseases.

## 1. Introduction

Liver cancer ranks ninth in incidence and fifth in mortality worldwide [1]. Hepatocellular carcinoma (HCC) is the most common primary liver tumor, accounting for 85–90% of diagnosed cases, and chronic liver disease is the most significant risk factor for the development of this tumor [2]. Hepatic carcinogenesis is a complex, multifactorial process involving various molecular mechanisms that occur throughout the multiple stages of chronic liver disease progression. Although the molecular etiology of HCC varies depending on specific causes and genotoxic damage, the most common changes include genetic and epigenetic alterations, inflammatory processes, oxidative stress responses, and immune responses [3].

The current therapeutic landscape for advanced HCC includes sorafenib, an oral first-line multikinase inhibitor; however, acquired resistance significantly limits its long-term clinical efficacy in a large proportion of patients [4,5]. Therefore, identifying key molecular targets in liver carcinogenesis represents a promising strategy for developing more effective therapies. To achieve this, our research group has explored drug repositioning and combination therapy as an innovative alternative for treating this type of cancer. Recently we evaluated the effect of combination therapy with loratadine, raloxifene, and sorafenib (LRS) on HepG2 and HuH7 liver cancer cell lines. This combination demonstrated significant synergy, inducing cytotoxicity in both cell lines [6].

The safety of these drugs and the significant antiproliferative effects of this combination make it a promising strategy for patients with HCC; therefore, elucidating the possible molecular mechanisms involved in its cytotoxicity could provide crucial information for the discovery of new therapeutic targets against cancer. Here, we characterized potential protein targets of loratadine, raloxifene, and sorafenib using the Drug Affinity Responsive Target Stability (DARTS) technique and molecular docking, identifying candidate drug targets with potential relevance to HCC and clinical prognosis. Notably, a substantial number of these proteins are involved in the hallmarks of cancer, and their expression levels strongly correlate with clinical outcomes and tumor behavior. The strong association between their expression levels and an adverse prognosis in patients with HCC highlights the clinical applicability of these findings, opening new opportunities for the identification of prognostic markers and molecular vulnerabilities amenable to pharmacological intervention in HCC and other cancers.

## 2. Results

### 2.1. Identification of Candidate DARTS-Stabilized Proteins of Loratadine, Sorafenib, and Raloxifene

To gain insights into the potential molecular mechanisms underlying the cytotoxic effect of the LRS combination previously described by our research group [6], we applied the DARTS technique to identify the pharmacological targets of the drugs (Figure 1a). We found 147 candidate targets for loratadine, 191 for sorafenib, and 157 for raloxifene (Figure 1b and Table 1). Supplementary Table S1 shows the complete list of identified proteins. These proteins included a wide variety of protein types, with enzymes as the most prevalent group of targets in all three cases (Figure 1c).

To further assess the possible drug–protein interactions identified by DARTS, three complementary computational and database-based approaches were implemented. Initially, an exploratory target prioritization strategy using virtual screening techniques—based on the generation of pharmacophore maps for each drug (Supplementary Figure S1)—allowed the identification of compounds with similar physicochemical properties. The hypothesis was that the molecular targets of compounds with physicochemical similarities are likely to be targets of the drugs of interest as well. Notably, an intersection of 31 proteins was identified in the loratadine group, 58 in the sorafenib group, and 7 in the raloxifene group across both approximations, providing a heuristic target space mapping between the DARTS and virtual screening approaches (Figure 2a,b). Table 2 shows examples of overlapping targets between the DARTS analysis and virtual screening for each drug; Supplementary Table S2 presents the total number of overlapped proteins in both strategies.

As a second approach to assess the binding potential of the drugs with some of the proteins identified with DARTS, we performed in silico molecular docking simulations.

The analysis focused on comparing the predicted binding scores (expressed in kcal/mol) of the candidate drugs with those of known reference ligands for each therapeutic target, as well as evaluating the specific intermolecular interactions stabilizing each putative complex (Figure 2c–e and Supplementary Figure S2). Docking scores for the candidate drugs were comparable to those obtained for the known reference ligands (Table 3). Figure 2c shows the predicted binding mode of loratadine to NGLY1, which displayed a more favorable docking score (Score = −7.4 kcal/mol) than the reference inhibitor Z-VAD-FMK (Score = −6.6 kcal/mol). The putative complex is stabilized via a conventional hydrogen bond with LYS A:530, complemented by electrostatic (π-cation/π-anion with LYS A:238 and GLU A:239), hydrophobic, and van der Waals interactions. Importantly, interactions with these specific amino acid residues have previously been proposed as key features for known NGLY1 inhibitors [7]. Sorafenib exhibited the most favorable predicted docking score with FKBP9 (Score = −8.0 kcal/mol), surpassing the reference ligand tacrolimus (Score = −7.7 kcal/mol), a macrolide that is well known to inhibit the catalytic activity of FKBP proteins. The observed interactions from the blind docking align with previous studies indicating that the sequence and topological diversity inherent to this multidomain protein family allows for versatile binding modes and varying affinities for a single ligand (Figure 2d) [8]. Raloxifene docked to the methyltransferase domain of THUMPD3 with a favorable predicted binding score (Score = −8.6 kcal/mol), outperforming the endogenous substrate S-adenosyl-L-methionine (SAM, Score = −8.0 kcal/mol). This targeted docking was directed at the active site, which was identified via structural alignment with the PDB entry 6ZXW (a methyltransferase co-crystallized with an SAM analog). In line with other methyltransferases, this active site features the conserved D[L/I]PFG signature motif. The putative raloxifene complex is stabilized by specific interactions with ASP A:406, PRO A:408, and PHE A:409 (Figure 2e)—residues known to be critical for the catalytic function of the enzyme [9].

Lastly, to additionally verify the interaction between the drugs and the experimentally identified proteins, we queried public databases—including ChEMBL, SwissTargetPrediction, PharmMapper, and DrugCLIP—for known targets of each compound. Several proteins identified with DARTS matched known targets reported in the databases for the corresponding drug. The integration of these reference datasets provides an exploratory target space mapping that shows a moderate degree of consensus with the empirical hits identified via the DARTS technique. The consistency observed between the identified candidate protein targets and the contained data on these platforms supports the biological plausibility of the drug–protein interactions. The set of all identified proteins is detailed in Supplementary Table S2. The results from the three described approaches strongly suggest the accurate drug–target interaction revealed with DARTS.

To evaluate target space concordance without overstating heuristic overlap, we conducted hypergeometric enrichment tests comparing DARTS candidates against virtual screening target profiles and external prediction databases (Supplementary Table S3). The virtual screening space yielded target recovery rates significantly exceeding random expectation for loratadine (FE = 31.45, *p* = 1.89 × 10^−37^), sorafenib (FE = 18.77, *p* = 6.06 × 10^−57^), and raloxifene (FE = 13.46, *p* = 9.93 × 10^−7^). Among external platforms, SwissTargetPrediction, PharmMapper, and ChEMBL showed marked enrichment, whereas DrugCLIP exhibited significant overlap only for raloxifene (*p* = 0.011).

### 2.2. Identified Candidate Targets of Loratadine, Sorafenib, and Raloxifene Are Involved in Carcinogenesis

To further investigate the biological significance of the proteins identified experimentally, a functional enrichment analysis was performed using the GO Biological Process (GO_BP), Reactome, and Kyoto Encyclopedia of Genes and Genomes (KEGG) databases (Figure 3a). This analysis revealed a wide variety of involved cellular processes. For loratadine’s candidate targets, there is a predominant enrichment of processes related to organelle localization, microtubule polymerization, and cytoskeletal organization. In the case of sorafenib, the most significant processes include the regulation of translational elongation, the regulation of protein stability, and vesicle transport in the Golgi apparatus, while for raloxifene there is a significant enrichment in processes involved in translation, amino acid metabolism, and the positive regulation of microtubule polymerization.

To determine the functional relevance of the identified proteins, protein–protein interaction (PPI) networks were constructed for each drug (Figure 3b). The analysis revealed that, individually, the three drugs interact with groups of proteins that form highly interconnected networks, with significant PPI enrichment values (*p*-value < 1 × 10^−16^). The presence of highly interconnected networks suggests that the three drugs modulate complex biological processes by simultaneously altering multiple proteins and cellular pathways.

The PPI network for loratadine demonstrated a strong convergence toward translation initiation and nucleolar dynamics, centered on key hub proteins highlighted in blue (specifically RPSA, RPS3, and NPM1), alongside intracellular vesicular trafficking regulators such as RAB11A. For raloxifene, the network highlighted central interactions focused on protein folding and ribosomal structural integrity, featuring prominent hubs such as the chaperonin subunit CCT3, the 40S ribosomal protein RPS5, and receptor tyrosine kinase signaling via EGFR. In contrast, sorafenib exhibited a densely interconnected core strongly enriched in protein quality control and cellular stress response pathways, driven primarily by VCP and HSPA8, in conjunction with scaffolding signaling hubs such as RACK1. Notably, all three drugs converge on shared or functionally overlapping nodes—specifically, ribosomal subunits (RPS3) and nucleolar regulators (NPM1). Given the high endogenous abundance of ribosomal machinery and chaperones in cell lysates, it is more appropriate to contextualize these shared nodes as a module of the proteostasis network with overlapping elements, rather than considering them to be independent drivers of synergy. Their joint involvement suggests the existence of a possible common axis of ribosomal or nucleolar stress that operates in parallel with the compound-specific target pathways.

We then evaluated whether the functions of the proteins identified above are related to carcinogenesis by assessing the association of these proteins with hallmarks of cancer. To do this, we used the consensus core proposed by Chen et al. [10], which establishes the relationship between hallmarks of cancer and genes using the GO_BP annotations. Based on this approach, we identified a group of proteins with annotations in biological processes linked to hallmarks of cancer. A full list of candidate targets mapped to each hallmark of cancer is detailed in Supplementary Table S4 (condensed summary shown in Table 4).

### 2.3. DARTS Identifies Candidate Drug Targets Associated with Prognostic Features in Hepatocellular Carcinoma

To determine whether the identified target proteins have clinical relevance in HCC patients, we investigated their association with clinical prognosis in terms of overall survival. Using the TCGA RNA-Seq database for liver hepatocellular carcinoma (LIHC), we determined the association between patient survival and mRNA expression of each protein through Kaplan–Meier survival curve analysis (Figure 4a). Among the identified proteins with available TCGA expression data, a statistically significant association with overall survival (FDR < 0.05) was confirmed in 20.14% of loratadine’s candidate targets, 22.93% for raloxifene, and 24.47% for sorafenib (Supplementary Table S5).

To establish whether the prognostic value of the identified proteins for each drug was independent of established clinical risk factors, a multivariate Cox proportional hazards regression analysis was performed, adjusting for tumor stage, histological grade, and patient age in the TCGA-LIHC cohort (Figure 4b). Among the identified proteins of the drugs, high expression of *KPNA2* (HR = 2.08, 95% CI: 1.39–3.11, *p* = 0.00034), *CCT3* (HR = 2.20, 95% CI: 1.47–3.29, *p* = 0.00012), *MYL6B* (HR = 1.97, 95% CI: 1.33–2.91, *p* = 0.00072), *CIT* (HR = 1.97, 95% CI: 1.33–2.93, *p* = 0.00079), *HSPH1* (HR = 1.91, 95% CI: 1.29–2.81, *p* = 0.0011), and *G6PD* (HR = 1.77, 95% CI: 1.2–2.63, *p* = 0.0043) independently predicted worse overall survival. Conversely, *CHP1* (HR = 0.55, 95% CI: 0.38–0.82, *p* = 0.0028) and *FOLH1* (HR = 0.61, 95% CI: 0.41–0.89, *p* = 0.011) were confirmed as independent factors associated with prolonged survival. Although a subpopulation of univariately significant genes lost independent prognostic capacity after controlling for clinical parameters (e.g., ALDOA, *p* = 0.0601; *RPS8*, *p* = 0.062), this multivariate evaluation identified a priority panel of candidate target proteins that maintain independent association with patient survival, providing a rational framework to investigate whether their pharmacological modulation by the proposed multi-drug regimen contributes to its therapeutic efficacy regardless of baseline tumor stage or grade.

To confirm that candidate targets identified by physical binding (DARTS-MS) translate to elevated protein expression in clinical samples, we evaluated protein-level expression patterns in the Human Protein Atlas comparing normal liver tissue and liver hepatocellular carcinoma (Supplementary Figure S3). Tissue proteomics profiling confirmed significant protein-level overexpression in tumor samples for *KPNA2* (*p* < 1 × 10^−30^), *G6PD* (*p* < 7 × 10^−12^), and *HSPH1* (*p* < 2 × 10^−34^); these protein-level comparisons confirm that a subset of top candidate targets is robustly overexpressed at the protein level in HCC tissue.

While the potential role of some of the identified proteins in liver carcinogenesis has been already investigated, we performed a PubMed literature search and found that the function of various candidate proteins has not yet been characterized in the context of liver cancer (Table 5). Furthermore, high mRNA expression levels of some of these proteins are associated with the clinical prognosis of patients with HCC (Figure 5 and Supplementary Figures S4–S6). To confirm that candidate targets identified by physical binding (DARTS-MS) translate to elevated protein expression in clinical samples, we evaluated protein-level expression patterns in the Human Protein Atlas comparing normal liver tissue and liver hepatocellular carcinoma (Supplementary Figure S3). Tissue proteomics profiling confirmed significant protein-level overexpression in tumor samples for *DOCK7* (*p* < 3 × 10^−20^), *RPN2* (*p* < 2 × 10^−17^), *FKBP9* (*p* < 9 × 10^−26^), and *THUMPD3* (*p* < 3 × 10^−34^); these protein-level comparisons confirm that a subset of top candidate targets is robustly overexpressed at the protein level in HCC tissue. In contrast, candidates such as *NGLY1* and *ICE1* exhibited non-significant protein expression trends. These findings open a new avenue for further investigation into liver carcinogenesis.

## 3. Discussion

Hepatocellular carcinoma remains one of the most lethal cancers worldwide, driven by a heterogeneous network of molecular mechanisms that contribute to its aggressiveness and chemoresistance. Despite the availability of targeted therapies, the clinical efficacy of current treatment options remains limited, emphasizing the need to identify molecular vulnerabilities in the tumor. To address this limitation, in this study, we employed the DARTS technique to map candidate protein targets of loratadine, raloxifene, and sorafenib, with the aim of deciphering the molecular basis underlying the cytotoxicity of this drug combination [6], while also identifying key and novel mediators in hepatocarcinogenesis.

The DARTS technique is a robust methodological tool for identifying protein targets of molecules of interest; its application is essential for elucidating undescribed mechanisms of action in the context of drug repositioning, particularly when reevaluating non-oncology agents. A recent study successfully employed this approach to uncover N-myristoyl transferase 1 (NMT1) as the primary target of desloratadine in HCC. This work revealed that desloratadine inhibits NMT1 activity, disrupting VILIP3 myristoylation and downstream NF-κB/Bcl-2 signaling to suppress tumor growth, and the authors highlighted NMT1 as a promising therapeutic target in HCC [11]. Using this approach, we identified a group of proteins with binding affinity for loratadine, raloxifene, and sorafenib. Notably, the expression of several of these proteins showed a direct correlation with overall survival in patients with HCC (Supplementary Table S5). Furthermore, GO annotations revealed that a significant fraction of these proteins actively participate in pathways associated with the hallmarks of cancer, suggesting potential relevance in hepatocarcinogenesis (Table 4).

A key observation of this study was the identification of undescribed potential candidate targets for these drugs, whose expression is significantly associated with clinical prognosis in patients with HCC, and whose role in liver carcinogenesis has not yet been elucidated (Figure 4 and Table 5). These results open new opportunities for exploring their function in liver cancer.

It is essential to discuss the biological implications of the identified proteins in order to contextualize their potential relevance in hepatocarcinogenesis. A deeper understanding of their function not only allows us to explain how they influence the progression of liver cancer but also helps to elucidate the signaling networks modulated by the drugs under evaluation. For instance, it is very interesting that the three drugs bind to cytoskeletal proteins that are relevant not only in cell division and migration but also in the reorganization of the phenotype of cancer cells. The complete analysis of the biological implications of most of the identified drug candidate targets deserves a comprehensive and extensive literature and database review. Nonetheless, based on their potential clinical relevance in HCC, we selected two examples to illustrate some of the feasible analysis of our findings, especially in those cases where the revealed proteins have not yet been associated with liver carcinogenesis. Below, the mechanistic and clinical roles of N-glycanase 1 (NGLY1) and ubiquitin recognition factor in ER-associated degradation 1 (UFD1) in cancer are discussed.

NGLY1/Png1 is essential for endoplasmic reticulum-associated protein degradation (ERAD), a critical process for preventing cytotoxicity caused by the accumulation of misfolded proteins [12]. Although recent evidence links NGLY1 activity to the pathogenesis of various diseases, including cancer [13], its direct involvement in HCC has not been fully characterized. In this study, DARTS identified NGLY1 as a candidate loratadine-interacting protein, and molecular docking provided computational support for a possible interaction. Furthermore, analysis of clinical and transcriptomic data from the TCGA revealed significant overexpression of NGLY1 mRNA in HCC tumor tissue, which is strongly associated with an unfavorable prognosis and lower overall survival in patients (Figure 4). The association between NGLY1 activity and carcinogenesis has been studied in melanoma, leukemia, and colorectal cancer [13,14,15]. In particular, NGLY1 has been proposed as a promising therapeutic target in the treatment of melanoma, given that its inhibition leads to cell-stress-mediated apoptosis [16]. Furthermore, NGLY1 inhibition has shown synergy with established therapeutic strategies, such as proteasome inhibition in multiple myeloma [17]. In this regard, the use of WRR139—a specific NGLY1 inhibitor—significantly enhanced the cytotoxicity of carfilzomib (a proteasome inhibitor used to treat multiple myeloma) [18].

At the molecular level, NGLY1 interacts with nuclear factor-erythroid-derived-2-related factor-1 (Nrf1), a key transcription factor in the proteotoxic stress response. Proteasome inhibition activates the Nrf1 pathway, resulting in the de novo synthesis of proteasome-related genes and the subsequent recovery of proteasome activity [19,20,21]. Several studies have demonstrated that the functional activation of Nrf1 depends on its NGLY1-mediated deglycosylation, a post-translational process critical for its transcriptional capacity [21,22]. In this context, NGLY1 deficiency compromises the cell’s ability to cope with proteotoxic stress. Collectively, blocking NGLY1 as a therapeutic strategy prevents the functional maturation of Nrf1, inhibiting both proteasome-mediated degradation and the effective activation of the autophagic pathway. As a result, the cell loses its two main pathways for the removal of damaged proteins—the ubiquitin–proteasome system (UPS), and the autophagy–lysosome pathway (ALP)—leading to the accumulation of cytotoxic protein aggregates, sustained cellular stress, and the induction of cell death.

In addition to its role in protein homeostasis, NGLY1 regulates cell survival under conditions of oxidative stress. NGLY1 promotes the expression of GPX4—an enzyme that is central to the neutralization of peroxidized lipids and, thus, confers resistance to ferroptosis—independently of NRF2 [23]. In parallel, NGLY1 exerts immunoregulatory functions in the tumor microenvironment by acting on key proteins such as intrinsic PD-1. In response to doxorubicin, NGLY1 promotes the deglycosylation and destabilization of PD-1, sensitizing tumor cells to the antiproliferative effects of the chemotherapeutic agent [24].

Taken together, this evidence positions NGLY1 as a key molecule in the adaptive capacity of tumor cells in response to various types of stress, and its inhibition represents a promising therapeutic strategy for various types of cancer, including HCC. Given that NGLY1 dysfunction is also associated with neurodegenerative diseases (such as Alzheimer’s and Parkinson’s), epilepsy, and liver disorders, our findings on the interaction between loratadine and NGLY1 not only support its therapeutic potential in oncology but also open new opportunities of research for the repurposing of this drug in other chronic diseases.

Sorafenib targets multiple kinases involved in carcinogenesis. Through the DARTS assay and molecular docking simulations, we determined that this drug exhibits affinity for the ubiquitin recognition factor in ER-associated degradation 1 (UFD1), which, in complex with VCP and NPLOC4, acts as a master regulator of proteostasis in the ERAD pathway; the activity of this complex has recently been linked to the determination of cell survival and fate in various types of cancer [25,26].

The role of UFD1 in carcinogenesis has been studied in various types of cancer; it promotes the progression of breast cancer and plays a significant role in colon cancer [27,28]. In breast cancer cells, blocking its activity promotes cell-cycle arrest in the G1 phase [29], underscoring its importance in cell proliferation. In the context of HCC, a recent study revealed that miR-3200—a molecule associated with tumorigenesis—negatively regulates its transcriptional capacity [30].

A critical aspect of UFD1 is its relationship with relevant oncogenes such as Myc. In T-cell acute lymphoblastic leukemia (T-ALL), Myc positively regulates UFD1 transcription; overexpression of this protein in human T-ALL cells induces endoplasmic reticulum (ER) stress, triggering apoptosis [31]. This finding suggests that UFD1 is a critical regulator of the ER stress response and a key determinant of the aggressiveness of Myc-driven leukemia. The authors suggest that targeting UFD1 constitutes a useful therapeutic strategy for Myc-driven cancers, given that many cases of liver cancer fall into this category [32]. These findings raise the possibility that UFD1 may represent a therapeutic pathway in selected cancer contexts.

The importance of modulating the activity of UFD1 in complex with VCP-NPLOC4 extends to its effects on the tumor microenvironment. In HCC, VCP has been reported to act as a key regulator in the suppression of CD8+ T cells, limiting their ability to generate antitumor responses [33]. Therefore, modulating the UFD1-VCP-NPLOC4 complex would not only impact the intrinsic protein homeostasis of the cancer cell but could also enhance the antitumor immune response, establishing itself as a useful therapeutic strategy in cancer treatment.

Although inhibiting the ubiquitin-dependent proteolytic pathway is a reasonable strategy for inducing cell death via proteotoxic stress in cancer, the activity of the UFD1-VCP-NPLOC4 complex has pleiotropic effects. Inhibiting the activity of this complex would trigger global protein stress that would affect both malignant and healthy cells. To overcome this limitation, Zhu et al. identified that phosphorylation at Ser784 of VCP (pSer784-VCP) is an event specific to DNA damage induced by genotoxic chemotherapy in patients with breast cancer. Interestingly, elevated levels of pSer784-VCP are significantly associated with poorer survival in patients treated with chemotherapy, suggesting that this event is implicated in treatment resistance [34]. An innovative strategy to avoid systemic toxicity would be to selectively block the interaction between UFD1 and the phosphorylated form of VCP in this patient population. However, further research is needed to determine whether this phosphorylation event occurs and has the same clinical relevance in other types of cancer, such as HCC.

Several technical and biological limitations inherent to our DARTS-MS discovery pipeline and proteomics workflow warrant discussion. First, whole-cell lysate preparation disrupts cellular compartmentalization and under-represents hydrophobic integral membrane proteins, such as canonical primary targets (e.g., GPCRs and receptor tyrosine kinases). This can be largely attributed to our sample preparation workflow, which utilized a hydrosoluble extraction protocol optimized for cytosolic and soluble protein fractions. Consequently, canonical receptors were not recovered in the proteomics dataset, and future studies employing membrane-specific fractionations and detergent-based solubilization buffers will be valuable to comprehensively map both membrane-bound canonical receptors and soluble intracellular network interactors simultaneously.

Second, proteolysis protection assays were conducted using drug concentrations equivalent to 10xIC_50_ in cell lysates. Although these elevated concentrations are widely utilized in DARTS workflows to compensate for non-specific protein binding in dense lysates and ensure detectable protease resistance, they exceed peak clinically achievable plasma levels and increase the propensity for non-specific biophysical stabilization.

Third, our target identification pipeline was not followed by direct biophysical or functional validation. Specifically, candidate targets identified via DARTS-MS were not independently confirmed using secondary orthogonal binding assays (e.g., concentration-dependent DARTS–Western blot, CETSA, or SPR to determine binding kinetics and affinities). Furthermore, while these candidate drivers are associated with poor clinical prognosis in HCC, loss- or gain-of-function experiments were not performed to directly test whether their perturbation mediates the cytotoxic, apoptotic, or synergistic pharmacological effects of the combination therapy.

Finally, our discovery pipeline was conducted exclusively in the HuH7 cell line, which cannot fully encompass the vast genomic and metabolic heterogeneity of human HCC. Future studies extending DARTS profiling to complementary cell lines (e.g., HepG2) and evaluating shared candidate interactors across diverse backgrounds will be essential to establish target robustness.

Taken together, our results provide a valuable experimental identification of candidate targets of loratadine, raloxifene, and sorafenib. The association of these proteins with clinical–pathological data emphasizes their potential as key molecules in hepatocarcinogenesis, opening a promising field for investigating their utility not only as tumor markers but also as priority targets in the development of therapeutic strategies against liver cancer.

## 4. Materials and Methods

### 4.1. Cell Culture and Reagents

The human liver cancer cell line HuH7 was obtained from the American Type Culture Collection (ATCC, Manassas, VA, USA) and cultured according to the manufacturer’s instructions. HuH7 cells were cultured in High-Glucose Dulbecco’s Modified Eagle Medium (DMEM) supplemented with 10% fetal bovine serum and 1% penicillin–streptomycin. Cells were maintained at 37 °C in a humidified atmosphere containing 5% CO_2_. The authenticity of the cell line was verified by STR genetic profiling in September 2014. The analysis showed the 12 alleles reported for the HuH-7 cell line, with 100.00% concordance. All experiments were performed using cells within low passage numbers (passages 6 to 13) to ensure phenotype stability.

For DARTS assays, the cells were cultured for 24 h in DMEM culture medium to further obtain the protein extract samples. The drugs (loratadine, raloxifene and sorafenib) were purchased from Sigma Aldrich Co. (St Louis, MO, USA). In all assays, the concentration of the vehicle (DMSO) was <1.56%.

### 4.2. Cell Lysate and Protein Extraction for DARTS Analysis

After incubating the cells for 24 h, the culture medium was removed, and the cells were washed three times with cold PBS. The cells were then harvested using a PBS-EDTA (0.03%) solution, and the cell suspension was transferred to a Falcon tube for centrifugation for 10 min at 3000 rpm and 4 °C. The PBS was removed from the resulting pellet, and the cells were resuspended in lysis buffer (M-PER) with protease inhibitors (20× Complete Mini, 200 mM orthovanadate, 500 mM NaF, and 200 nM PMSF) added; the cells were then incubated on ice for 30 min, with agitation every 5 min. After this time, the cell lysates were centrifuged at 12,000 rpm for 20 min at 4 °C; the supernatant (extracted proteins) was then transferred to a new tube, and 10X TNC buffer was added. The samples were stored at −80 °C until further analysis. Protein concentrations were determined using the BCA kit (Cat. No. 23250, Thermo Fisher, Waltham, MA, USA), and protein integrity was assessed by gel electrophoresis.

### 4.3. DARTS Assay

The DARTS technique was performed as described by Lomenick et al. (2011) and Pai et al. (2015) [35,36], with minor modifications. For each DARTS assay, 200 μL of the protein pool (∽2 μg/μL) was transferred to Eppendorf tubes, to which the drugs (loratadine, raloxifene, or sorafenib; 10x their IC_50_ obtained in our previous study [6]) (final micromolar concentrations: 86 for sorafenib; 252 for raloxifene; 300 for loratadine) or the vehicle (DMSO) was added individually, and the samples were incubated in a Thermomix for 4 h at 37 °C with agitation. After the incubation period, each condition was divided into two equal 100 μL fractions—a pronase-treated fraction (1 mg/mL, or 1:10 ratio) and a matched untreated control fraction—as illustrated in the experimental workflow schematic (Supplementary Figure S7). Digestion was maintained for 15 min at 37 °C under constant shaking and stopped by adding Complete Mini 20×. Then, the samples were centrifuged on centrifugation columns for 20 min at 12,000 *g* and 4 °C. The sample retained on the filter was collected and analyzed by mass spectrometry.

### 4.4. Identification of Target Proteins by Mass Spectrometry

For qualitative proteomics analysis, 100 μg of protein per replicate (n=3 independent biological replicates per condition) was processed. Enzymatic digestion was performed using the “in-Stage Tip” (iST) sample preparation protocol (PreOmics, Munich, Germany), with minor modifications [37,38]. Briefly, protein pellets were lysed in LYSE reagent at 95 °C for 10 min, sonicated for 20 cycles (30 s ON/30 s OFF) in a Bioruptor Pico (Diagenode), and digested with a Lys-C/Trypsin mix (“DIGEST” reagent) at 37 °C for 2 h. The resulting peptides were washed on iST cartridges (WASH 1 and WASH 2 buffers), eluted, evaporated to dryness in a SpeedVac concentrator, and resuspended in LC-LOAD reagent prior to analysis. Tryptic peptides were separated on an ACQUITY UPLC M-Class system equipped with an HSS T3 C18 column (Waters Corp, Milford, MA, USA; 75 μm × 150 mm, 100 A° pore size, 1.8 μm particle size), using 0.1% FA in H2O as mobile phase A and 0.1% FA in ACN as mobile phase B, under the following gradient: 7% B (0 min), 40% B (121.49 min), 85% B (123.15–126.46 min), and 7% B (129–130 min), at a flow of 400 nL·min−1 and 45 °C. Spectral data were acquired on a Synapt G2-Si mass spectrometer (Waters Corp, Milford, MA, USA) operating in positive nano-ESI mode using ion mobility separation and a Data-Independent Acquisition (DIA) approach via ${\mathrm{HDMS}}^{E}$ mode. Collision-induced dissociation (CID) fragmentation was applied using a 19–55 eV energy ramp, and internal calibration was maintained using [Glu1]-Fibrinopeptid.

Raw MS/MS files were processed for qualitative protein identification using ProteinLynx Global Server (PLGS v3.0.3, Waters Corp) against the Homo sapiens UniProt database [39,40] (UP0000005640, downloaded 27 February 2024; 104,557 sequences), using a target–decoy strategy. The search parameters allowed trypsin cleavage with one missed cleavage; carbamidomethylation (C) as a fixed modification; and amidation (N-term), deamidation (N, Q), oxidation (M), and phosphorylation (S, T, Y) as variable modifications. Identification stringency was set to a false discovery rate (FDR) ≤1%, requiring a minimum of 2 fragment ions per peptide, 5 fragment ions per protein, and at least 1 unique peptide per protein. All false-positive identifications (reversed proteins) were removed from the study, and the filtered protein results were exported to *.xlsx files for subsequent analysis.

### 4.5. Virtual Screening

Based on the generation of pharmacophore maps, a virtual screening of the DrugBank database was performed, and the identified targets were compared with those experimentally identified using the DARTS technique. For loratadine, two pharmacophore maps were used: one generated from structurally and pharmacologically related ligands (astemizole, mepyramine, and desloratadine), and another based on crystallographic structures available in the PDB (8 × 5Y, 8 × 63, and 8 × 64). For raloxifene, a pharmacophore map generated from related ligands (tamoxifen, levormeloxifene, estradiol, elacestrant, and arzoxifene) was used. For sorafenib, a pharmacophore map was also generated based on structurally and pharmacologically related ligands (axitinib, pazopanib, dacomitinib, erlotinib, and lapatinib).

### 4.6. Molecular Docking

The crystal structures of the reported proteins were retrieved from the Protein Data Bank and prepared by removing ligands and water molecules; structures not reported in the Protein Data Bank were generated using AlphaFold 3 [41]. Subsequently, the Dock Prep module of UCSF Chimera [42] was used to repair residues, add hydrogen atoms, and assign partial charges. The coordinates and dimensions of the docking boxes were defined to encompass the sites of interest, thereby ensuring that molecular docking explored the key interactions between the ligands and target proteins. Molecular docking was performed using AutoDock Vina (version 1.1.2) [43], whereas the visualization and analysis of the molecular interactions were carried out using Discovery Studio.

From the total number of proteins identified by the DARTS assay, a subset of six candidate targets was prioritized for molecular docking analysis. Protein selection was strictly conducted prior to docking based on two main criteria: a lack of prior characterization regarding their specific role in liver carcinogenesis, and poor clinical prognosis associated with high mRNA expression in the TCGA-LIHC cohort. No other targets were subjected to molecular docking.

### 4.7. Bioinformatic Analysis

To assess the extent of scientific evidence regarding the role of the identified target proteins in liver carcinogenesis, a systematic literature search was conducted in the PubMed database.

The clinical value of the selected molecules was determined by constructing Kaplan–Meier survival curves for overall patient survival using the TCGA PanCancer Atlas dataset on HCC (https://www.cbioportal.org/study/summary?id=lihc_tcga_pan_can_atlas_2018 (accessed on 08 March 2026)). Patients in the TCGA-LIHC cohort were dichotomized into high- and low-expression groups using a uniform median cutoff (50% high vs. 50% low) across all candidate genes. We deliberately avoided optimized cutpoints, in order to eliminate selection bias (*p*-hacking) and over-optimization.

Functional enrichment analyses for protein targets of the drugs identified by DARTS were conducted in the R statistical environment (version 4.5.2) using RStudio. To identify treatment-induced functional alterations, over-representation analysis (ORA) was performed on the proteins identified by DARTS using the clusterProfiler and ReactomePA packages, mapping differentially expressed molecules against the Gene Ontology (GO), Kyoto Encyclopedia of Genes and Genomes (KEGG), and Reactome databases.

Protein–protein interaction (PPI) networks for the identified proteins were constructed using Cytoscape software (version 3.10.4); the interaction data were retrieved from the STRING database (Search Tool for the Retrieval of Interacting Genes; https://string-db.org/ (accessed on 14 June 2026)).

## 5. Conclusions

Our findings identify potential clinically relevant novel targets of loratadine, raloxifene, and sorafenib in HCC and support their characterization as multitarget drugs. The association between the mRNA expression levels of the novel targets and adverse prognosis in patients with HCC highlights the clinical applicability of these findings, which open promising opportunities for future research into the identified interactome.

The DARTS findings were further supported by concordance with cross-validation using virtual screening techniques based on the generation of pharmacophore maps, in silico molecular docking simulations, and analysis from public databases (including ChEMBL, SwissTargetPrediction, PharmMapper, and DrugCLIP), showing that several known targets of each compound matched those identified in the results of our DARTS experiments.

However, a primary limitation of this study is that candidate target proteins identified through the DARTS-MS assay were not independently confirmed via secondary orthogonal binding assays (e.g., surface plasmon resonance to evaluate binding kinetics). While some of the proteins that we identified via DARTS have already been reported as targets of the studied drugs (supporting our findings), and virtual screening, molecular docking, and clinical prognostic analyses provide complementary target prioritization, in silico modeling cannot completely exclude potential proteomic or enzymatic artifacts inherent to mass spectrometry-based DARTS. Consequently, the targets reported herein should be interpreted as prioritized candidate interactors within the hepatocellular carcinoma network, serving as a framework for future formal biophysical and structural binding assays. Furthermore, while these target identification strategies successfully prioritize candidate proteins associated with poor prognosis, functional target validation through loss- and gain-of-function studies remains necessary in order to definitively confirm their mechanistic roles. Moreover, evaluating the functional phenotype of targets with uncharacterized roles in the development and progression of liver cancer offers a plethora of potential research regarding their involvement in early pre-tumorigenic stages, as well as their potential use as tumor markers. Furthermore, it would be very interesting to investigate the individual drug targets and their synergistic contributions to the observed cytotoxic phenotype, alongside the characterization of their expression profiles in tissue samples from animal models and HCC patients. While previous functional studies established the synergistic efficacy of the LRS combination, the present work focused on deconvoluting the baseline candidate targets of the individual drugs; demonstrating their significant target convergence provides a mechanistic framework for their synergy. Nevertheless, executing parallel multi-omics and DARTS analyses directly on cells exposed to the simultaneous LRS combination remains a key direction for future research to capture emergent multi-drug target networks. In addition, performing DARTS assays using the simultaneous three-drug mixture represents an interesting opportunity to evaluate potential physical cooperativity or synergism.

This approach provides new opportunities for the identification of novel targets, prognostic markers, and molecular vulnerabilities suitable for pharmacological intervention in HCC and other malignancies and diseases. However, because these candidate proteins were identified in HuH7 cells, they may not completely generalize across all HCC models, highlighting the need to evaluate these candidate interactions and their functional consequences in additional HCC backgrounds.

## Figures and Tables

**Figure 1:**
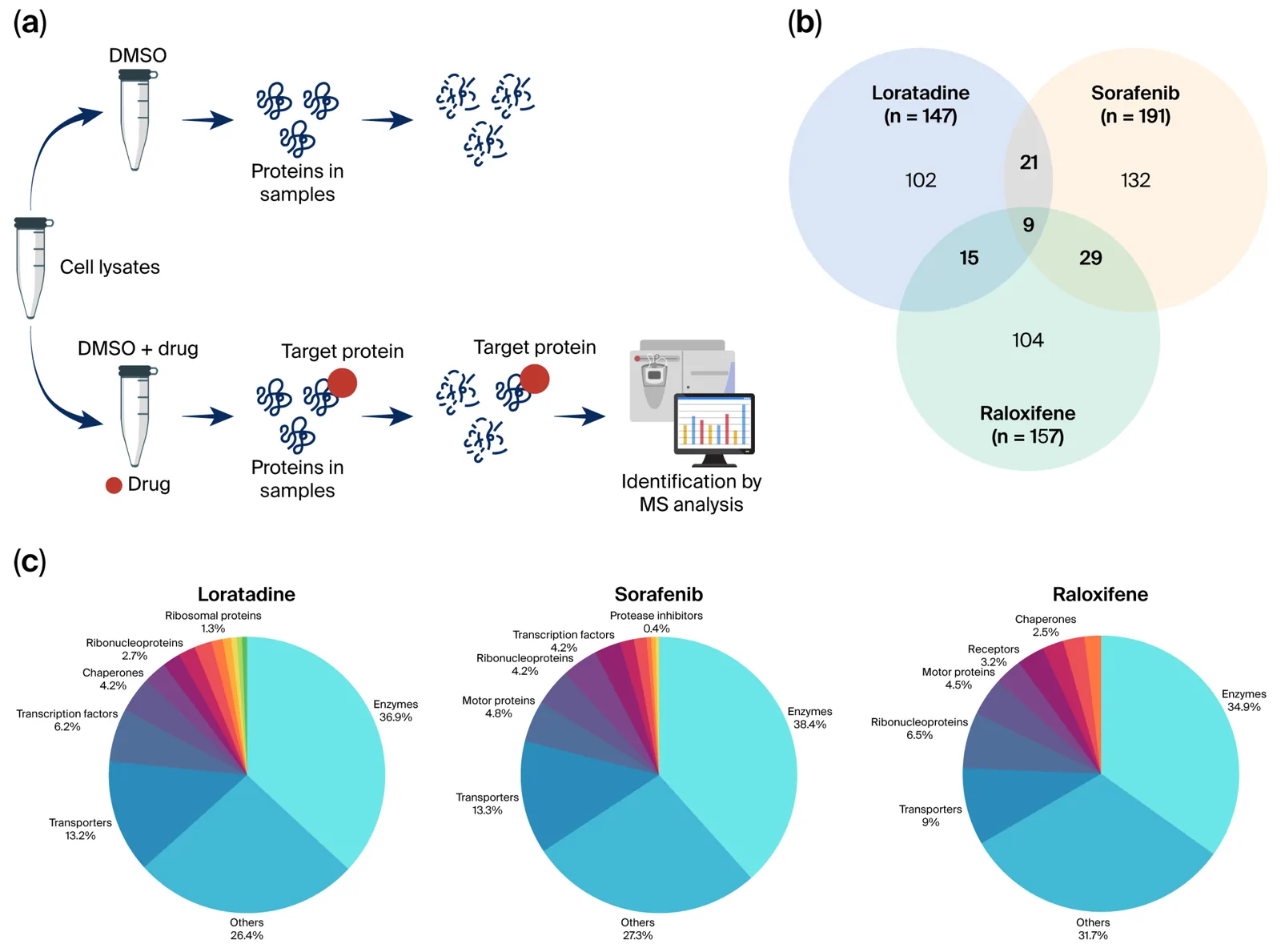
Identification of candidate protein targets of loratadine, raloxifene, and sorafenib by DARTS technique. (**a**) DARTS analysis methodology. (**b**) Total number of proteins for the three drugs identified experimentally. (**c**) Types of identified proteins in the DARTS assay.

**Figure 2:**
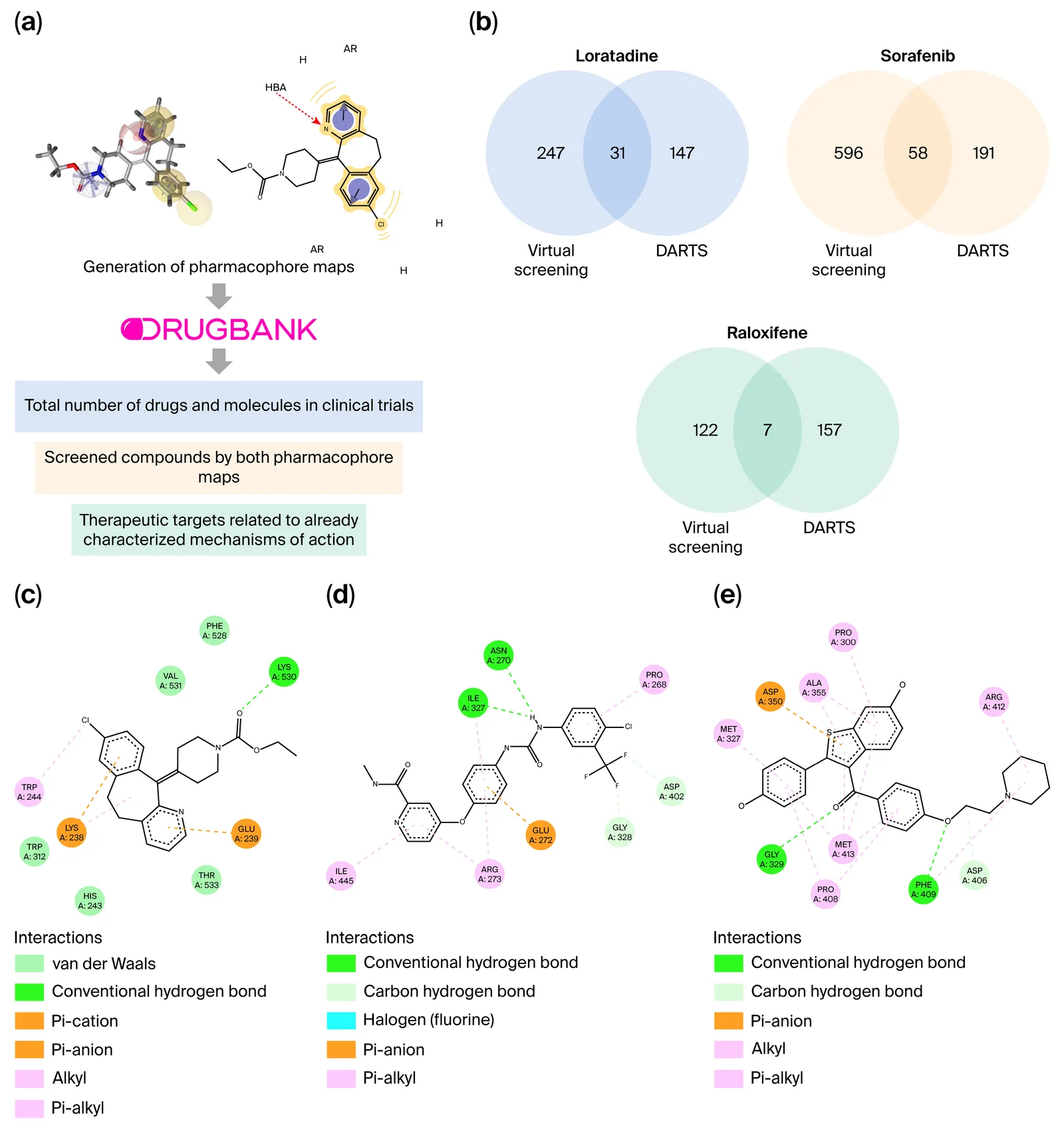
Pharmacophore-based virtual screening strategy for drug candidate target identification. (**a**) Methodology for virtual screening. (**b**) Number of target proteins identified in the DARTS analysis that correspond to previously reported drug targets. (**c**) Molecular docking targeting NGLY1, (**d**) FKBP9, and (**e**) THUMPD3.

**Figure 3:**
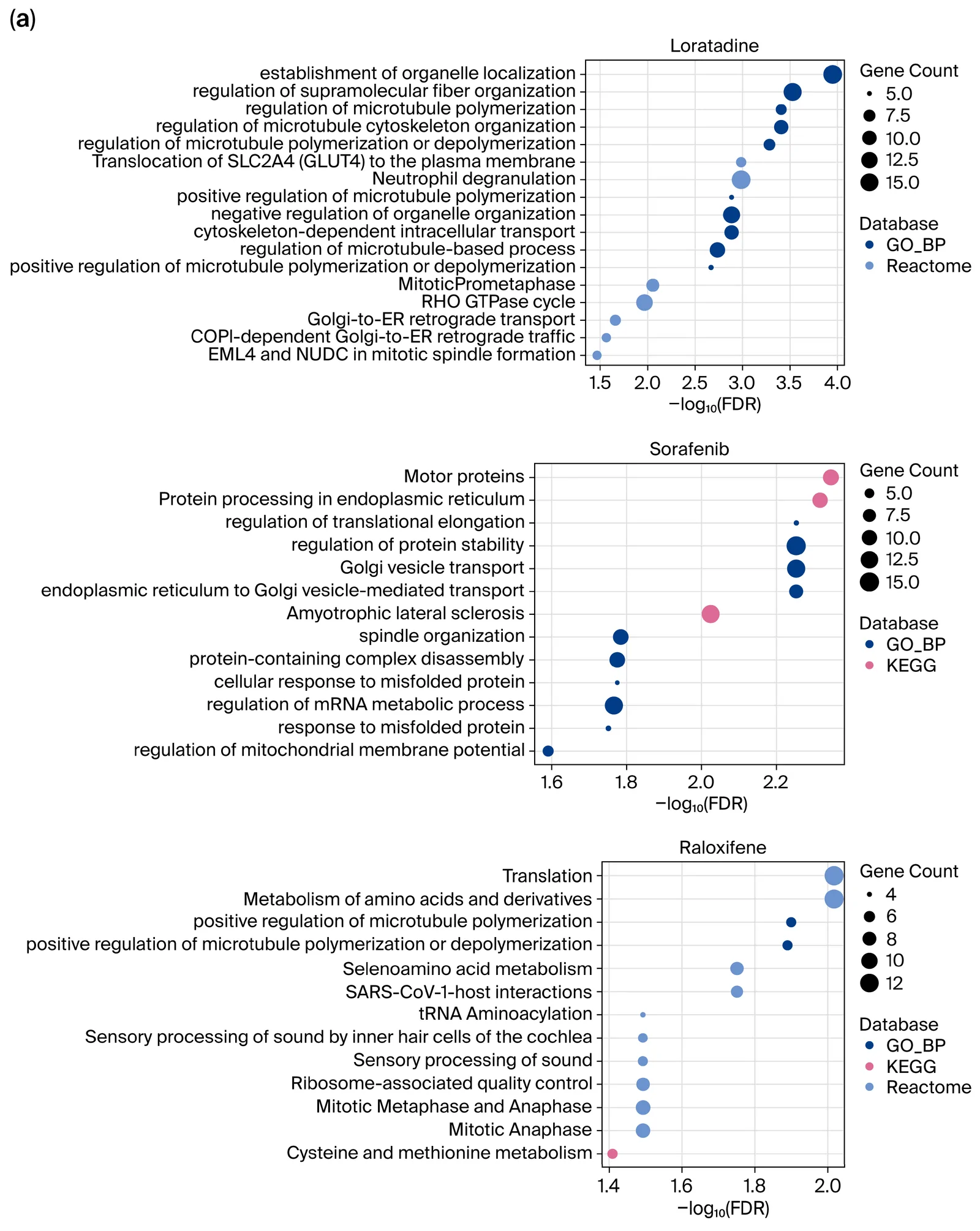

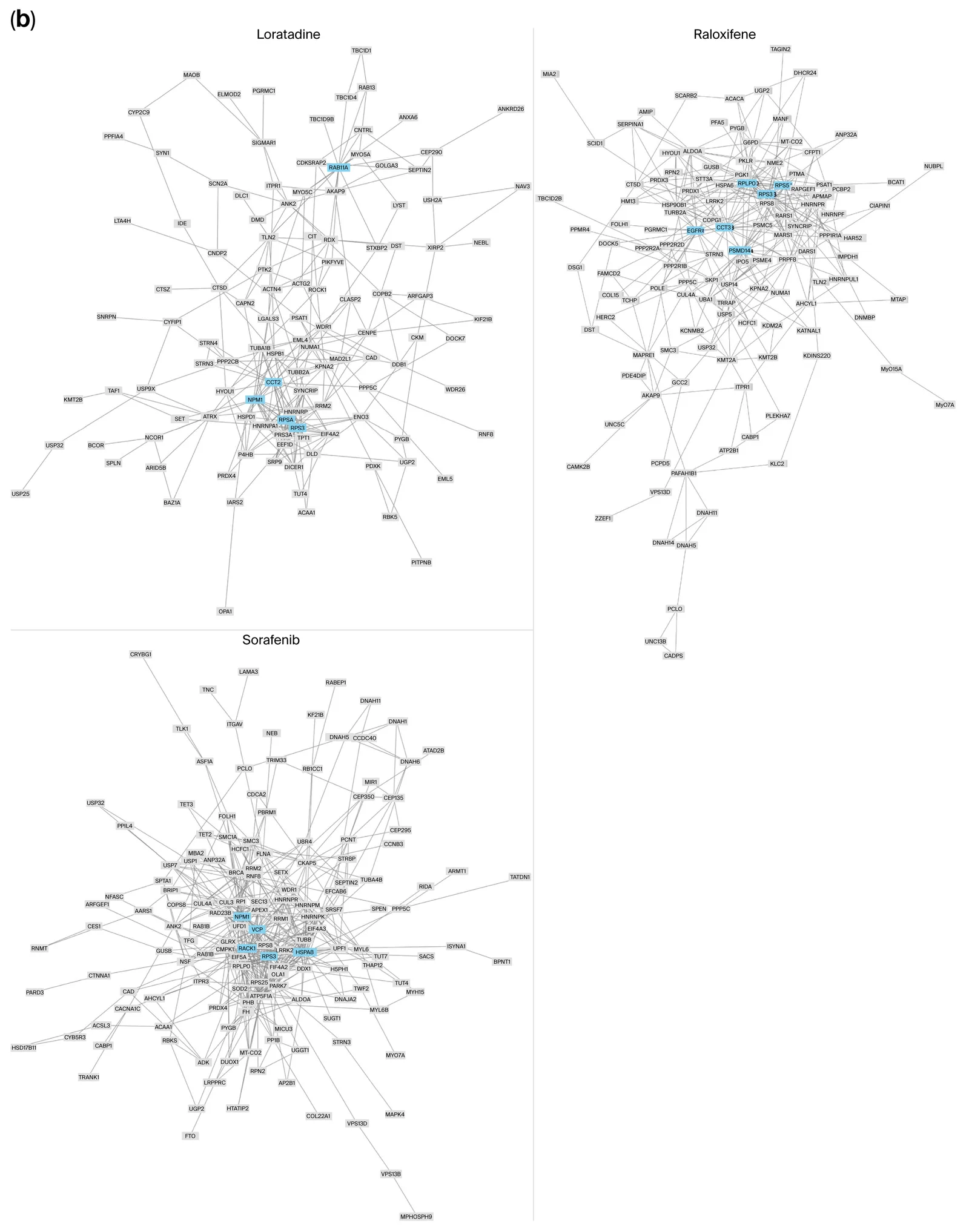
Functional enrichment analysis and characterization of the protein interactome of loratadine, sorafenib, and raloxifene candidate targets identified by DARTS. (**a**) Enriched biological pathways and cellular processes for the target proteins of each drug. The data are sourced from the GO_BP, Reactome, and KEGG databases; (**b**) PPI networks of candidate targets for loratadine, sorafenib and raloxifene, constructed using Cytoscape (version 3.10.4). Each node represents a protein, and the edges indicate functional interactions between them. The nodes in blue correspond to the proteins with the highest degree, that is, those with the greatest number of connections within the network and, therefore, considered central nodes with potential functional relevance in the mechanisms of action of the compounds.

**Figure 4:**
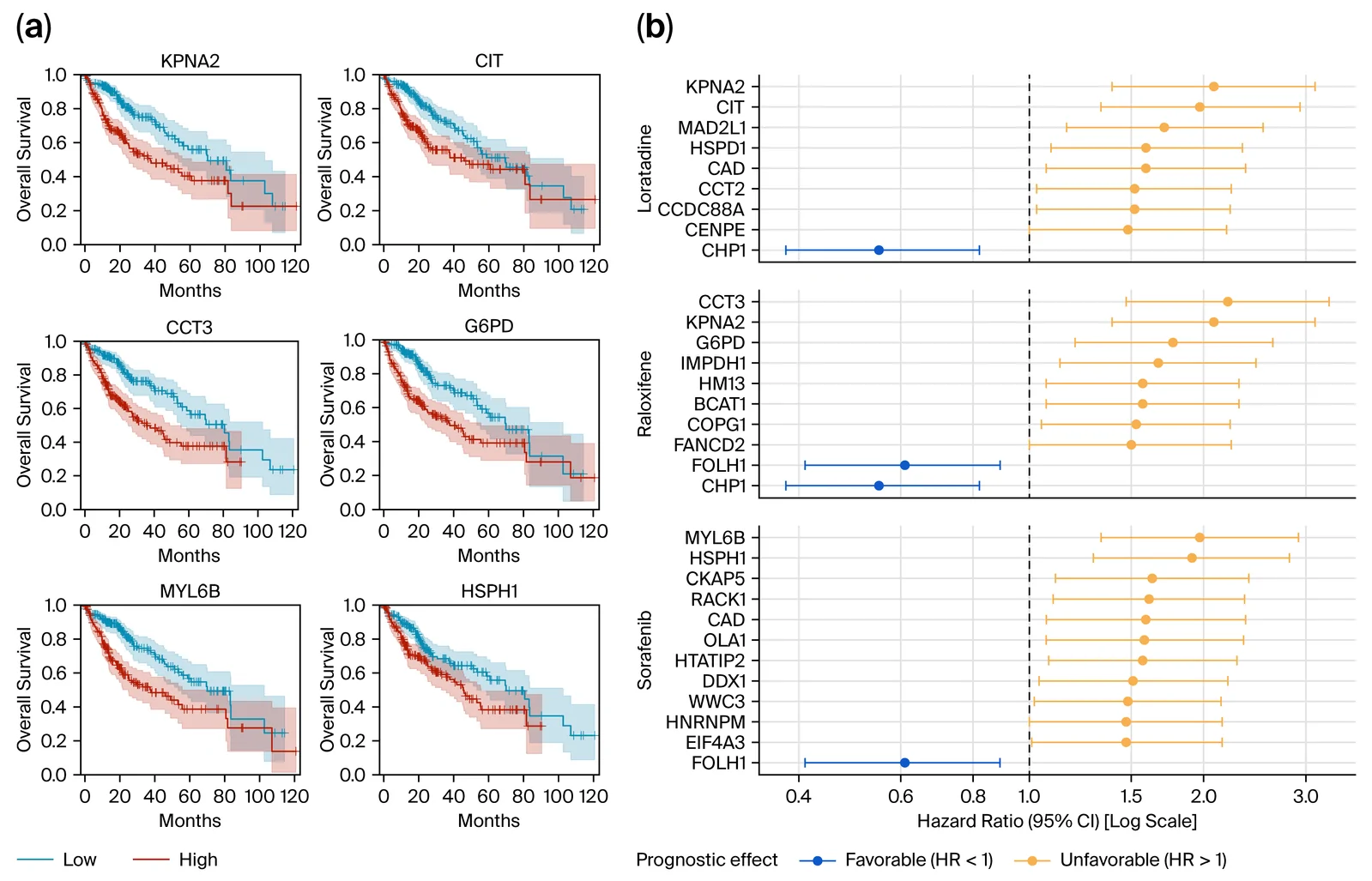
Kaplan–Meier overall survival curves and multivariate Cox proportional hazards regression of candidate target genes in hepatocellular carcinoma. (**a**) Kaplan–Meier overall survival curves for representative candidate targets comparing high (red) versus low (blue) expression groups in HCC patients (data from TCGA-LIHC project). Shaded areas represent 95% confidence intervals, and tick marks indicate censored observations. (**b**) Forest plot displaying multivariate Cox proportional hazards regression models adjusted for AJCC clinical stage, histological grade, and patient age, faceted by drug entity (loratadine, raloxifene, and sorafenib). Point estimates of Hazard Ratios (HR) and 95% confidence intervals (95% CI) are plotted on a logarithmic scale. Red markers denote independent unfavorable prognostic factors (HR > 1.0, *p* < 0.05), while green markers represent independent favorable prognostic factors (HR < 1.0, *p* < 0.05).

**Figure 5:**
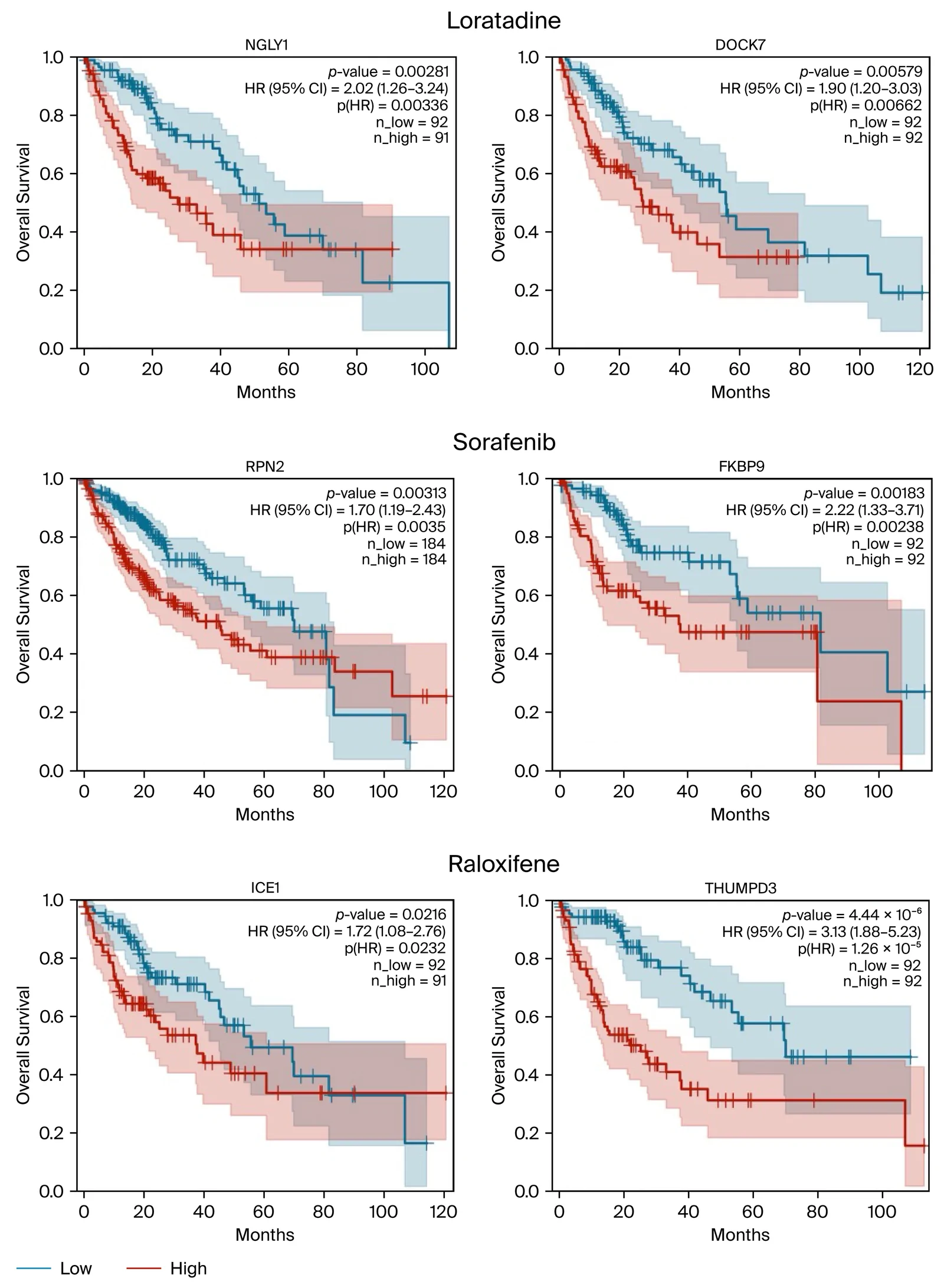
Kaplan–Meier curves of potential novel candidate targets for loratadine, raloxifene, and sorafenib in hepatocellular carcinoma. Kaplan–Meier curves display the 10-year overall survival based on mRNA expression levels of proteins identified via DARTS with uncharacterized roles in liver carcinogenesis. Data were obtained from the TGCA-LIHC project.

**Table 1:** Examples of target proteins identified for each drug using the DARTS technique.

Loratadine			Sorafenib			Raloxifene		
UniProt ID	Protein Name	Gene Symbol	UniProt ID	Protein Name	Gene Symbol	UniProt ID	Protein Name	Gene Symbol
P09651	Heterogeneous nuclear ribonucleoprotein A1	HNRNPA1	Q96J65	ATP-binding cassette sub-family C member 12	ABCC12	P00533	Epidermal growth factor receptor	EGFR
O75335	Liprin-alpha-4	PPFIA4	Q9UQE7	Structural maintenance of chromosomes protein 3	SMC3	P28340	DNA polymerase epsilon catalytic subunit	POLD1
P63267	Actin_ gamma-enteric smooth muscle	ACTG2	Q96JP2	Unconventional myosin-XVB	MYO15B	P07339	Cathepsin D	CTSD
P41219	Peripherin	PRPH	F8W1I5	Myosin light chain 6B	MYL6B	Q8NB902	ATPase family gene 2 protein homolog A	AFG2A
Q9Y4L1	Hypoxia up-regulated protein 1	HYOU1	P05388	Large ribosomal subunit protein uL10	RPLP0	P54578	Ubiquitin carboxyl-terminal hydrolase 14	USP14
P06748	Nucleophosmin	NPM1	P11216	Glycogen phosphorylase_ brain form	PYGB	P45974	Ubiquitin carboxyl-terminal hydrolase 5	USP5
Q9UBR2	Cathepsin Z	CTSZ	Q5S007	Leucine-rich repeat kinase 2	LRRK2	Q8NHM5	Lysine-specific demethylase 2B	KDM2B
P07237	Protein disulfide-isomerase	P4HB	Q9Y2K3	Myosin-15	MYO15A	Q8NFA0	Ubiquitin carboxyl-terminal hydrolase 32	USP32
Q15907	Ras-related protein Rab-11B	RAB11B	Q13402-5	Isoform 5 of Unconventional myosin-VIIa	MYO5A	Q9Y2K7	Lysine demethylase 2A	KDM2A
Q9Y4I1	Isoform 2 of Unconventional myosin-Va	MYO5A	O60884	DnaJ homolog subfamily A member 2	DNAJA2	Q15392	Delta(24)-sterol reductase	DHCR24

**Table 2:** Examples of proteins identified via DARTS and virtual screening approach.

Loratadine			Sorafenib			Raloxifene		
UniProt ID	Gene Symbol	Protein Name	UniProt ID	Gene Symbol	Protein Name	UniProt ID	Gene Symbol	Protein Name
Q9Y4L1	HYOU1	Hypoxia up-regulates protein 1	Q9H0U4	RAB1B	Ras-related protein Rab-1B	P00533	EGFR	Epidermal growth factor receptor
Q9UBR2	CTSZ	Cathepsin Z	Q93009	USP7	Ubiquitin carboxyl-terminal hydrolase 7	P28340	POLE	DNA polymerase epsilon catalytic subunit
Q15907	RAB11B	Ras-related protein Rab-11B	Q96J65	ABCC12	ATP-binding cassette sub-family C member 12	Q9UKL3	CASP8AP2	Caspase 8 associated protein 2
P62136	PPP1CA	Serine/threonine-protein phosphatase	P62136	PPP1CA	Serine/threonine-protein phosphatase	Q15392	DHCR24	Delta(24)-sterol reductase
P51153	RAB13	Ras-related protein Rab-13	Q9UQE7	SMC3	Structural maintenance of chromosomes protein 3	Q9Y2K7	KDM2A	Lysine demethylase 2A
P07339	CTSD	Cathepsin D	P11216	PYGB	Glycogen phosphorylase	P07339	CTSD	Cathepsin D
P06401	PGR	Progesterone receptor	Q69YH5	CDCA2	Cell division cycle-associated protein 2	P43034	PAFAH1B1	Platelet activating factor acetylhydrolase 1b
Q99250	SCN2A	Sodium channel protein type 2	P55263	ADK	Adenosine kinase	Q99250	SCN2A	Sodium channel protein type 2
P27338	MAOB	Amine oxidase B	O60884	DNAJA2	DnaJ homolog subfamily A member 2	Q03164	KMT2A	Histone-lysine N-methyltransferase 2A
Q8NFA0	USP32	Ubiquitin carboxyl-terminal hydrolase 32	Q14240	EIF4A2	Eukaryotic initiation factor 4A-II	Q8NB90	ATAD2	ATPase family gene 2 protein homolog A

**Table 3:** Molecular docking parameters of candidate protein targets evaluated with loratadine, sorafenib, and raloxifene.

Protein	Source of Structure	Drug and Score (kcal/mol)	Reference Compound and Score (kcal/mol)
NGLY1	AlphaFold	Loratadine (−7.4)	Z-VAD-FMK (−6.6)
BCOR	PDB 8HCU	Loratadine (−6.6)	CAS 879790-53-1 (−8.8)
FKBP9	AlphaFold	Sorafenib (−8.0)	Tacrolimus (−7.7)
TUT4	PDB 6IW6	Sorafenib (−7.4)	TS-002266 (−7.7)
UFD1	PDB 5B6C	Sorafenib (−8.0)	NA
THUMPD3	AlphaFold	Raloxifene (−8.6)	SAM (−8.0)

**Table 4:** Gene Ontology annotations for the candidate targets of the three drugs associated with hallmarks of cancer.

Hallmark of Cancer	Biological Process (GO Terms)	Number of Identified Proteins	Representative Proteins
Sustaining proliferative signaling	Cell cycle, cell division, cell population proliferation, negative regulation of cell cycle,	95	ATRX, BCAT1, BRCA2, BRIP1, CASP8AP2
Evading growth suppressor	Negative regulation of cell growth, negative regulation of cell population proliferation	53	G6PD, PHB, RACK1, TCHP
Resist cell death	Negative regulation of programmed cell death, programmed cell death,	70	HTATIP2, HYOU1, ITGAV, KDM2B, LGALS3
Enabling replicative immortality	Cellular senescence, regulation of telomere maintenance, telomere maintenance	10	ATRX, CCT2, CCT3, HNRNPA1, PARP1, UPF1, USP7
Inducing Angiogenesis	Angiogenesis, positive regulation of angiogenesis, vasculogenesis	7	COL22A1, FLNA, HTATIP2, ITGAV, MYDGF, PTK2
Activating Invasion and Metastasis	Cell adhesion, cell junction organization, cell migration, cell projection organization	93	MYOT, NFASC, PARD3, PCLO, PFN2, PLEKHA7, PPFIA2, PPFIA4
Genome Instability and Mutation	DNA integrity checkpoint signaling, DNA repair	35	CUL4A, DDB1, DDX1, FANCD2, FH, FTO, HDGFL2, HERC2
Tumor promoting Inflammation	Regulation of inflammatory response	5	FANCD2, LRRK2, NLRP5, PARK7, TNC
Deregulating Cellular Energetic	Cellular response to hypoxia, Glycolytic process	8	ALDOA, ENO3, BRIP1, HYOU1, P4HB, PGK1, SCN2A, PKLR
Avoiding Immune Destruction	Immune response, Regulation of immune response	18	LYST, PCBP2, PHB, PLCL2, PRDX1, PRSS3, PTK2, RNF8, STXBP2, SYNCRIP, TUBB

**Table 5:** Drug candidate targets with potential relevance to hepatocellular carcinoma identified using DARTS and not yet characterized in the context of liver carcinogenesis.

Drug	Uniprot ID	Gene	Prognostic Value in HCC
Loratadine	Q96IV03	*NGLY1*	Unfavorable *
	Q96N67	*DOCK7*	Unfavorable *
	Q9NRL22	*BAZ1A*	Unfavorable
	Q5TAX3	*TUT4*	Unfavorable *
	Q6W2J9	*BCOR*	Unfavorable
Sorafenib	P048442	*RPN2*	Unfavorable
	Q5TAX3	*TUT4*	Unfavorable *
	Q92890	*UFD1*	Unfavorable
	O95302	*FKBP9*	Unfavorable *
	Q92734	*TFG*	Unfavorable
	P52758	*RIDA*	Favorable
Raloxifene	Q9Y2F5	*ICE1*	Unfavorable *
	P048442	*RPN2*	Unfavorable
	P56192	*MARS1*	Unfavorable
	P49590	*HARS2*	Unfavorable
	Q9BV44	*THUMPD3*	Unfavorable *
	Q9Y678	*COPG1*	Unfavorable

* Prognostic value (overall survival) reached statistical significance (*p* < 0.05) when evaluating extreme expression cohorts (top 25% vs. bottom 25% expression quartiles), whereas standard median-cutoff stratifications showed non-significant trends, suggesting a threshold-dependent clinical impact in LIHC patients.

## Data Availability

The analyzed and raw data from the current study are available from the corresponding author upon request.

## Supplementary Materials

The following supporting information can be downloaded at: https://www.mdpi.com/article/10.3390/ijms27198648/s1.

## Abbreviations

DARTS	Drug Affinity Responsive Target Stability
ERAD	Endoplasmic Reticulum-Associated Protein Degradation
GO	Gene Ontology
HCC	Hepatocellular carcinoma
HPA	Human protein atlas
KEGG	Kyoto Encyclopedia of Genes and Genomes
LIHC	Liver Hepatocellular Carcinoma
LRS	Loratadine, raloxifene and sorafenib
NGLY1	N-glycanase 1
PPI	Protein–protein interaction
SAM	S-adenosyl-L-methionine
TCGA	The Cancer Genome Atlas
UFD1	Ubiquitin Recognition Factor in ER-associated Degradation 1
UPS	Ubiquitin–Proteasome System

## References

[1] Global Cancer Observatory. https://gco.iarc.who.int/.

[2] Suddle A., Reeves H., Hubner R., Marshall A., Rowe I., Tiniakos D., Hubscher S., Callaway M., Sharma D., See T.C. (2024). British Society of Gastroenterology Guidelines for the Management of Hepatocellular Carcinoma in Adults. Gut.

[3] Panneerselvam S., Wilson C., Kumar P., Abirami D., Pamarthi J., Reddy M.S., Varghese J. (2023). Overview of Hepatocellular Carcinoma: From Molecular Aspects to Future Therapeutic Options. Cell Adhes. Migr..

[4] Ladd A.D., Duarte S., Sahin I., Zarrinpar A. (2024). Mechanisms of Drug Resistance in HCC. Hepatology.

[5] Tang W., Chen Z., Zhang W., Cheng Y., Zhang B., Wu F., Wang Q., Wang S., Rong D., Reiter F.P. (2020). The Mechanisms of Sorafenib Resistance in Hepatocellular Carcinoma: Theoretical Basis and Therapeutic Aspects. Signal Transduct. Target. Ther..

[6] Villarruel-Melquiades F., Hernandez-Gallegos E., Solano-Agama C., Mendoza-Garrido M.E., Camacho J. (2023). The Combination Sorafenib-Raloxifene-Loratadine as a Novel Potential Therapeutic Approach Against Human Liver Cancer. In Vivo.

[7] Liu R., Gu J., Ye Y., Zhang Y., Zhang S., Lin Q., Yuan S., Chen Y., Lu X., Tong Y. (2023). A Natural Compound Containing a Disaccharide Structure of Glucose and Rhamnose Identified as Potential N-Glycanase 1 (NGLY1) Inhibitors. Molecules.

[8] Somarelli J.A., Lee S.Y., Skolnick J., Herrera R.J. (2008). Structure-based Classification of 45 FK506-binding Proteins. Proteins.

[9] Wang C., Tay L.L.E., Hu W., Corre M., Graille M. (2025). TRMT112, a Master Activator of Several Methyltransferases Modifying Factors Involved in RNA Maturation and Translation. Front. RNA Res..

[10] Chen Y., Verbeek F.J., Wolstencroft K. (2021). Establishing a Consensus for the Hallmarks of Cancer Based on Gene Ontology and Pathway Annotations. BMC Bioinform..

[11] Tan X.-P., He Y., Yang J., Wei X., Fan Y.-L., Zhang G.-G., Zhu Y.-D., Li Z.-Q., Liao H.-X., Qin D.-J. (2023). Blockade of NMT1 Enzymatic Activity Inhibits N-Myristoylation of VILIP3 Protein and Suppresses Liver Cancer Progression. Signal Transduct. Target. Ther..

[12] Krshnan L., Van De Weijer M.L., Carvalho P. (2022). Endoplasmic Reticulum–Associated Protein Degradation. Cold Spring Harb. Perspect. Biol..

[13] Zhan C., Zhao X., Wei Y., Yang R., Yan N. (2025). Unveiling Roles of NGLY1 in Cellular Homeostasis and Related Diseases. Cell. Signal..

[14] Oliveira T., Zhang M., Joo E.J., Abdel-Azim H., Chen C.-W., Yang L., Chou C.-H., Qin X., Chen J., Alagesan K. (2021). Glycoproteome Remodeling in MLL-Rearranged B-Cell Precursor Acute Lymphoblastic Leukemia. Theranostics.

[15] Yepes S., Tucker M.A., Koka H., Xiao Y., Jones K., Vogt A., Burdette L., Luo W., Zhu B., Hutchinson A. (2020). Using Whole-Exome Sequencing and Protein Interaction Networks to Prioritize Candidate Genes for Germline Cutaneous Melanoma Susceptibility. Sci. Rep..

[16] Zolekar A., Lin V.J., Mishra N.M., Ho Y.Y., Hayatshahi H.S., Parab A., Sampat R., Liao X., Hoffmann P., Liu J. (2018). Stress and Interferon Signalling-Mediated Apoptosis Contributes to Pleiotropic Anticancer Responses Induced by Targeting NGLY1. Br. J. Cancer.

[17] Kegyes D., Gulei D., Drula R., Cenariu D., Tigu B., Dima D., Tanase A., Badelita S., Buzoianu A.-D., Ciurea S. (2023). Proteasome Inhibition in Combination with Immunotherapies: State-of-the-Art in Multiple Myeloma. Blood Rev..

[18] Tomlin F.M., Gerling-Driessen U.I.M., Liu Y.-C., Flynn R.A., Vangala J.R., Lentz C.S., Clauder-Muenster S., Jakob P., Mueller W.F., Ordoñez-Rueda D. (2017). Inhibition of NGLY1 Inactivates the Transcription Factor Nrf1 and Potentiates Proteasome Inhibitor Cytotoxicity. ACS Cent. Sci..

[19] Lehrbach N.J., Ruvkun G. (2016). Proteasome Dysfunction Triggers Activation of SKN-1A/Nrf1 by the Aspartic Protease DDI-1. eLife.

[20] Vangala J.R., Radhakrishnan S.K. (2019). Nrf1-Mediated Transcriptional Regulation of the Proteasome Requires a Functional TIP60 Complex. J. Biol. Chem..

[21] Ward M.A., Vangala J.R., Kamber Kaya H.E., Byers H.A., Hosseini N., Diaz A., Cuervo A.M., Kaushik S., Radhakrishnan S.K. (2024). Transcription Factor Nrf1 Regulates Proteotoxic Stress-Induced Autophagy. J. Cell Biol..

[22] Lehrbach N.J., Breen P.C., Ruvkun G. (2019). Protein Sequence Editing of SKN-1A/Nrf1 by Peptide:N-Glycanase Controls Proteasome Gene Expression. Cell.

[23] Forcina G.C., Pope L., Murray M., Dong W., Abu-Remaileh M., Bertozzi C.R., Dixon S.J. (2022). Ferroptosis Regulation by the NGLY1/NFE2L1 Pathway. Proc. Natl. Acad. Sci. USA.

[24] Wu D., Wu Z., Yao H., Yan X., Jiao Z., Liu Y., Zhang M., Wang D. (2024). Doxorubicin Induces Deglycosylation of Cancer Cell-intrinsic PD-1 by NGLY 1. FEBS Lett..

[25] Fujisawa R., Polo Rivera C., Labib K.P. (2022). Multiple UBX Proteins Reduce the Ubiquitin Threshold of the Mammalian P97-UFD1-NPL4 Unfoldase. eLife.

[26] Yuan C., Liao Y., Si W., Huang M., Li D., Wang F., Quan Y., Yu X., Liao S. (2025). Trim21 Modulates Endoplasmic Reticulum-Associated Degradation and Sensitizes Cancer Cells to ER Stress-Induced Apoptosis by Inhibiting VCP/Npl4/UFD1 Assembly. Biochim. Biophys. Acta (BBA)-Mol. Basis Dis..

[27] Chen M., Gutierrez G.J., Ronai Z.A. (2011). Ubiquitin-Recognition Protein Ufd1 Couples the Endoplasmic Reticulum (ER) Stress Response to Cell Cycle Control. Proc. Natl. Acad. Sci. USA.

[28] Wang X., Guo Q., Wang H., Yuan X., Wang B., Lobie P.E., Zhu T., Tan S., Wu Z. (2021). *PCBP2* Posttranscriptional Modifications Induce Breast Cancer Progression via Upregulation of UFD1 and NT5E. Mol. Cancer Res..

[29] Shi X., Zhu K., Ye Z., Yue J. (2020). VCP/P97 Targets the Nuclear Export and Degradation of P27^Kip1^ during G1 to S Phase Transition. FASEB J..

[30] Song S., Xie S., Liu X., Li S., Wang L., Jiang X., Lu D. (2023). miR-3200 Accelerates the Growth of Liver Cancer Cells by Enhancing Rab7A. Non-Coding RNA Res..

[31] Huiting L., Samaha Y., Zhang G., Roderick J., Li B., Anderson N., Wang Y., Wang L., Laroche F., Choi J. (2018). UFD1 Contributes to MYC-Mediated Leukemia Aggressiveness through Suppression of the Proapoptotic Unfolded Protein Response. Leukemia.

[32] Liu F., Liao Z., Zhang Z. (2023). MYC in Liver Cancer: Mechanisms and Targeted Therapy Opportunities. Oncogene.

[33] Cheng C., Zha Q., Sun L., Cui T., Guo X., Xing C., Chen Z., Ji C., Liang S., Tao S. (2025). VCP Downstream Metabolite Glycerol-3-Phosphate (G3P) Inhibits CD8+T Cells Function in the HCC Microenvironment. Signal Transduct. Target. Ther..

[34] Zhu C., Rogers A., Asleh K., Won J., Gao D., Leung S., Li S., Vij K.R., Zhu J., Held J.M. (2020). Phospho-Ser784-VCP Is Required for DNA Damage Response and Is Associated with Poor Prognosis of Chemotherapy-Treated Breast Cancer. Cell Rep..

[35] Lomenick B., Jung G., Wohlschlegel J.A., Huang J. (2011). Target Identification Using Drug Affinity Responsive Target Stability (DARTS). CP Chem. Biol..

[36] Pai M.Y., Lomenick B., Hwang H., Schiestl R., McBride W., Loo J.A., Huang J., Hempel J.E., Williams C.H., Hong C.C. (2015). Drug Affinity Responsive Target Stability (DARTS) for Small-Molecule Target Identification. Chemical Biology.

[37] Avalos-Gómez C., Aguilar-Chaparro M.A., Ríos-Castro E., Marin-Flamand E., Sánchez-Mendoza A.E., De La Garza M. (2026). Differences between Protein Cargo in Outer Membrane Vesicles Released from *Mannheimia haemolytica* A2 in the Presence and Absence of Bovine Lactoferrin. J. Proteom..

[38] Ortega-Lozano A.J., Jiménez-Uribe A.P., Aranda-Rivera A.K., Gómez-Caudillo L., Ríos-Castro E., Tapia E., Bellido B., Aparicio-Trejo O.E., Sánchez-Lozada L.G., Pedraza-Chaverri J. (2022). Expression Profiles of Kidney Mitochondrial Proteome during the Progression of the Unilateral Ureteral Obstruction: Focus on Energy Metabolism Adaptions. Metabolites.

[39] Käll L., Storey J.D., MacCoss M.J., Noble W.S. (2008). Assigning Significance to Peptides Identified by Tandem Mass Spectrometry Using Decoy Databases. J. Proteome Res..

[40] Elias J.E., Gygi S.P., Hubbard S.J., Jones A.R. (2010). Target-Decoy Search Strategy for Mass Spectrometry-Based Proteomics. Proteome Bioinformatics.

[41] Abramson J., Adler J., Dunger J., Evans R., Green T., Pritzel A., Ronneberger O., Willmore L., Ballard A.J., Bambrick J. (2024). Accurate Structure Prediction of Biomolecular Interactions with AlphaFold 3. Nature.

[42] Pettersen E.F., Goddard T.D., Huang C.C., Couch G.S., Greenblatt D.M., Meng E.C., Ferrin T.E. (2004). UCSF Chimera—A Visualization System for Exploratory Research and Analysis. J. Comput. Chem..

[43] Trott O., Olson A.J. (2010). AutoDock Vina: Improving the Speed and Accuracy of Docking with a New Scoring Function, Efficient Optimization, and Multithreading. J. Comput. Chem..

